# Wearable sensors during drawing tasks to measure the severity of essential tremor

**DOI:** 10.1038/s41598-022-08922-6

**Published:** 2022-03-28

**Authors:** Sheik Mohammed Ali, Sridhar Poosapadi Arjunan, James Peters, Laura Perju-Dumbrava, Catherine Ding, Michael Eller, Sanjay Raghav, Peter Kempster, Mohammod Abdul Motin, P. J. Radcliffe, Dinesh Kant Kumar

**Affiliations:** 1 RMIT University, Melbourne, VIC Australia; 2 SRM Institute of Science and Technology, Chennai, TN India; 3 Monash Health, Clayton, VIC Australia

**Keywords:** Neurology, Biomedical engineering

## Abstract

Commonly used methods to assess the severity of essential tremor (ET) are based on clinical observation and lack objectivity. This study proposes the use of wearable accelerometer sensors for the quantitative assessment of ET. Acceleration data was recorded by inertial measurement unit (IMU) sensors during sketching of Archimedes spirals in 17 ET participants and 18 healthy controls. IMUs were placed at three points (dorsum of hand, posterior forearm, posterior upper arm) of each participant’s dominant arm. Movement disorder neurologists who were blinded to clinical information scored ET patients on the Fahn–Tolosa–Marin rating scale (FTM) and conducted phenotyping according to the recent Consensus Statement on the Classification of Tremors. The ratio of power spectral density of acceleration data in 4–12 Hz to 0.5–4 Hz bands and the total duration of the action were inputs to a support vector machine that was trained to classify the ET subtype. Regression analysis was performed to determine the relationship of acceleration and temporal data with the FTM scores. The results show that the sensor located on the forearm had the best classification and regression results, with accuracy of 85.71% for binary classification of ET versus control. There was a moderate to good correlation (*r*^2^ = 0.561) between FTM and a combination of power spectral density ratio and task time. However, the system could not accurately differentiate ET phenotypes according to the Consensus classification scheme. Potential applications of machine-based assessment of ET using wearable sensors include clinical trials and remote monitoring of patients.

## Introduction

Essential tremor (ET), which affects roughly 2% of the population, is the commonest cause of tremor in neurological practice^1,2^. Research into the disorder depends strongly on objective methods to characterise the tremor and to measure its severity. In 2013, a task force established by the Movement Disorder Society to review rating scales for the assessment of ET recommended 5 of these scales^3^. While each is a valid clinical tool, the report draws attention to weaknesses of reproducibility and of floor and ceiling effects. Further development of automated measurement of tremor severity may lead to more precise evaluation of treatment effects in ET clinical trials.

Screening of ET, for verification of diagnosis, and for inclusion in population-based studies, is a further research application of clinical scales. A challenge for any ET clinical diagnostic instrument is the lack of a ‘gold standard’, and ET may in fact be several rather than one disease entity. Unlike the second commonest tremor disorder, which is Parkinson’s disease (PD), it lacks a distinct pathological basis. There are grey areas—with normal or physiological tremor in mild cases, and with disorders such as dystonic tremor^4^ and tremor-dominant PD^5^. The recent Consensus Statement on the Classification of Tremors^6^ has attempted to address some of these uncertainties by sub-classifying ET syndromes. ET is separated into ET and ET plus, the additional ‘plus’ criteria encompassing patients who meet basic criteria but have, in addition, ‘soft’ findings such as impaired tandem gait, questionable dystonic posturing, memory impairment or other mild neurological signs that do not suffice to diagnose another syndrome^7^. Analysis of tremor frequency and electromyographic activity can help in the diagnosis of ET. Automated tests may also prove useful in identifying ‘minor motor’ features that distinguish ET plus.

Technological advancement has led to the development of motion sensors that are wireless and wearable. Acceleration^8–10^, gyroscopic^11,12^ and electromyographic^13,14^ data can all be recorded. Research groups^15–20^ have proposed the use of such technology to detect and monitor parkinsonian and essential tremors. Multiple sensing modules have been used for data collection from separate anatomical locations^21,22^. A single wrist-worn accelerometer for the assessment of rest tremors and bradykinesia has also been proposed for PD^23^. ET shows a frequency between 4 and 12 Hz; its variable amplitude depends on factors such as anxiety, limb position, voluntary activity and disease duration^20^. Clinical measurements may be performed at rest, with the arms held in posture, or with goal-directed or repetitive tasks^20,24^. Some sensor systems have significant shortcomings: limited accuracy with prolonged measurement, and a need for axial calibration. While devices such as Fitbit or smartphone watch apps can estimate total tremor activity, the proprietary data of such devices are not well suited to diagnosing or monitoring tremor disorders.

Clinicians use pen and paper tests in the diagnosis and monitoring of ET^25^. Spiral drawing is a particularly suitable task for ET. It entails continuous movement in multiple planar directions rather than the more up-and-down actions of writing^26,27^. The commonly used Fahn-Tolosa-Marin scale (FTM)^28^ has a domain devoted to the analysis of drawing Archimedes spirals, as does the Bain and Findley scale^29^. Digital drawing tablet analysis of spiral drawing has been shown to be more sensitive to small changes in tremor severity than visual spiral rating methods^30^. They do, however, have the limitation that only 2-dimensional activity is captured.

This work proposes a method to record and analyse movement data captured during Archimedes spiral drawing by three-axis accelerometers embedded in inertial movement unit (IMU) devices. The chief study aims were twofold—to validate the estimation of the clinical tremor severity score from the sensor recordings; and to classify ET data with respect to the Consensus Statement scheme for ET^6^. A secondary aim was to report on suitable positions for placement of sensors to record upper limb tremor signal. We processed these signals to combine the three acceleration axes into a single vector magnitude^23^. This was then analysed to obtain power spectral density (PSD) for two frequency bands: 0.5—4 Hz and 4 -12 Hz. Most voluntary movement occurs in the lower band, while the upper band captures tremor, both physiological and pathological^31–33^. The ratio of the PSD between the two bands overcomes the need for normalisation and allows comparison between different people. We hypothesise that the ratio of PSD, along with the total time taken by the individual to perform the task of sketching a pre-defined Archimedes spiral, will correlate with the tremor severity measure FTM. To validate this, we employed statistical analysis and machine learning to establish the relationships of sensor features with clinical ratings and classifications.

## Results

Table 1 shows demographic information on the 35 participants (18 control, 17 ET) with analysable recordings. Eleven had been classified as ET plus; five had 1 plus feature (ET + 1) and 6 had 2 plus features (ET + 2). Six subjects fulfilled the Consensus Axis 1 definition of ET, henceforth abbreviated as ET − 0. The columns of Table 1 correspond to controls, ET − 0, ET + 1 and ET + 2. Demographic, clinical and sensor-derived statistics are shown. ET plus patients were older, and older at tremor onset, though the groups were broadly matched for the duration of ET. Though FTM tremor severity was similar for ET − 0 and ET + 1 groups, ET + 2 was associated with significantly higher scores. The inter-rater analysis for FTM scores showed a very strong correlation between the two blinded assessors (*r*^2^ = 0.95), supporting the choice of the FTM score as the clinical severity standard for this study. The estimated FTM scores from the Sensor-2 data appear below the clinical measurements. Cognition according to the MoCA was similar across the groupings. The measured sensor parameter of mean PSD ratios from the three sections of the arm (Sensor-1, Sensor-2, Sensor-3) and the mean task times are displayed in the bottom part of the table.

**Table 1:** The demographic, clinical and estimated sensors information and the P-value of the Kruskal–Wallis test between the groups of controls, ET − 0, ET + 1, ET + 2.

	Control	ET − 0	ET + 1	ET + 2	**P value**
Number	18	6	5	6	**–**
M:F	8:10	3:3	1:4	4:2	**–**
Age	62.8 ± 11.6	62.8 ± 16.5	61.4 ± 11.6	77.2 ± 7.3	**0.084**
Age at tremor onset	–	37.7 ± 18.5	51.2 ± 13.1	44.0 ± 25.9	**0.433**
Tremor duration	–	25.2 ± 23.2	10.2 ± 5.8	31.3 ± 20.5	**0.229**
MoCA	–	25.8 ± 4.2	25.2 ± 3.3	25.7 ± 2.8	**0.879**
Average group clinical FTM score	–	16.0 ± 9.2	20.4 ± 3.8	36.25 ± 12.24	**0.011**
Average group sensor estimated FTM score	_	19.63 ± 5.4	24.0 ± 10.5	29.59 ± 10.5	**0.197**
Mean PSD ratio (sensor-1)	0.55 ± 0.37	2.15 ± 1.66	3.90 ± 2.59	4.74 ± 1.82	**0.001**
Mean PSD ratio (sensor-2)	0.79 ± 1.12	2.84 ± 2.42	5.16 ± 5.59	6.96 ± 6.24	**0.002**
Mean PSD ratio (sensor-3)	0.81 ± 0.60	0.88 ± 1.14	0.97 ± 0.95	2.09 ± 2.59	**0.000**
Mean time	12.64 ± 5.53	20.53 ± 14.23	19.82 ± 8.04	26.92 ± 14.95	**0.007**

Significance values are in Bold.

**Figure 1:**
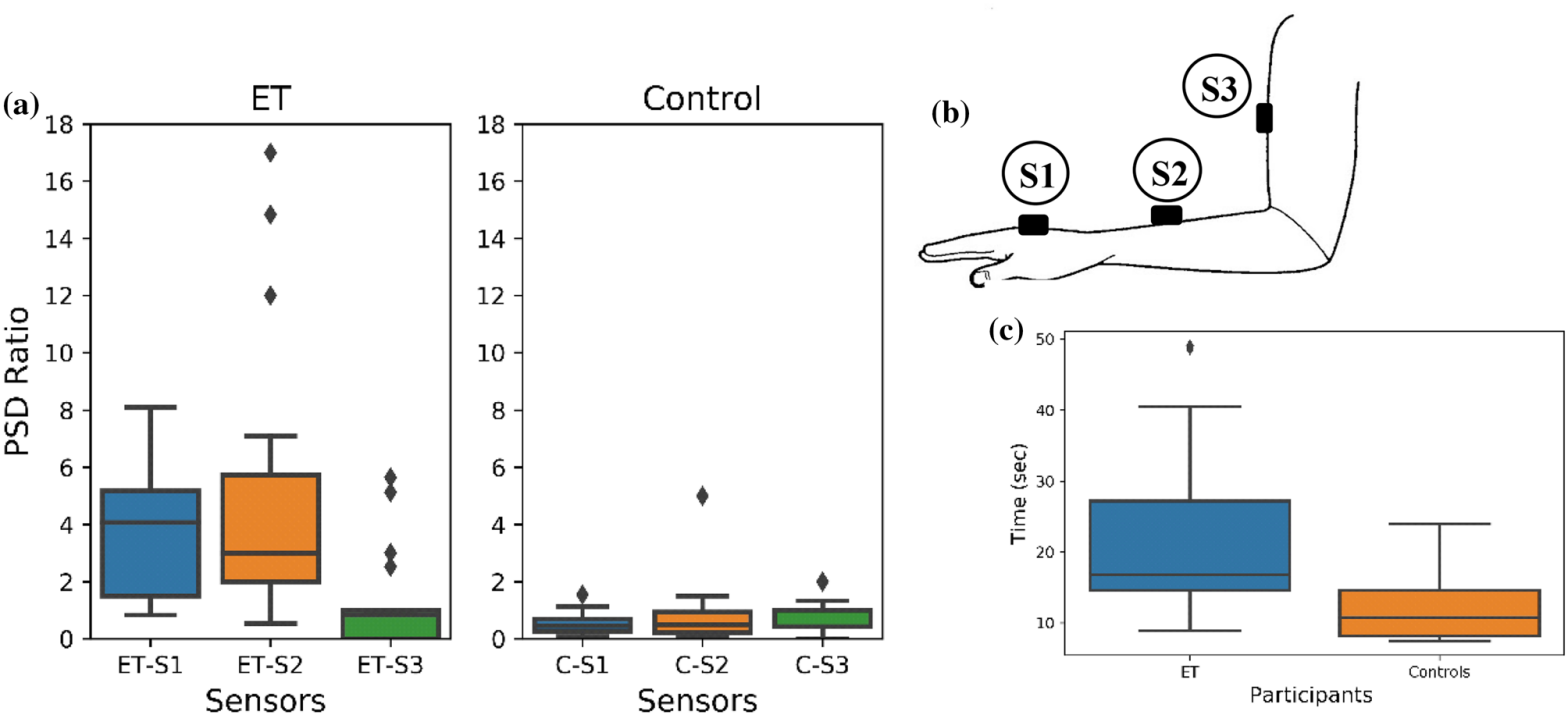
**a** Boxplot of the PSD ratio for ET and controls at the three sensing locations. ETS1—ET Sensor—1, ETS2—ET Sensor—2, ETS3—ET Sensor—3; and for Controls, CS1—Control Sensor—1, CS2—Control Sensor—2, CS3—Control Sensor—3 . (**b**) Shows the placement of the sensor location, S1, S2 and S3. (**c**) Boxplot of the Task Time of ET and controls at the sensing locations.

Figure 1 shows the box plot of the PSD ratio in ET and control groups measured from the three sensor locations illustrated in Fig. 5. The mean PSD ratio is higher in ET compared with controls for all three sensors.

Mann–Whitney U testing showed significant differences between ET and controls for the PSD ratio (*U* = 25.00, *p* = 0.00) for sensor-2, and for T (*U* = 52.00,* p* = 0.001), the task execution time.

### Estimation of ET severity

**Figure 2:**
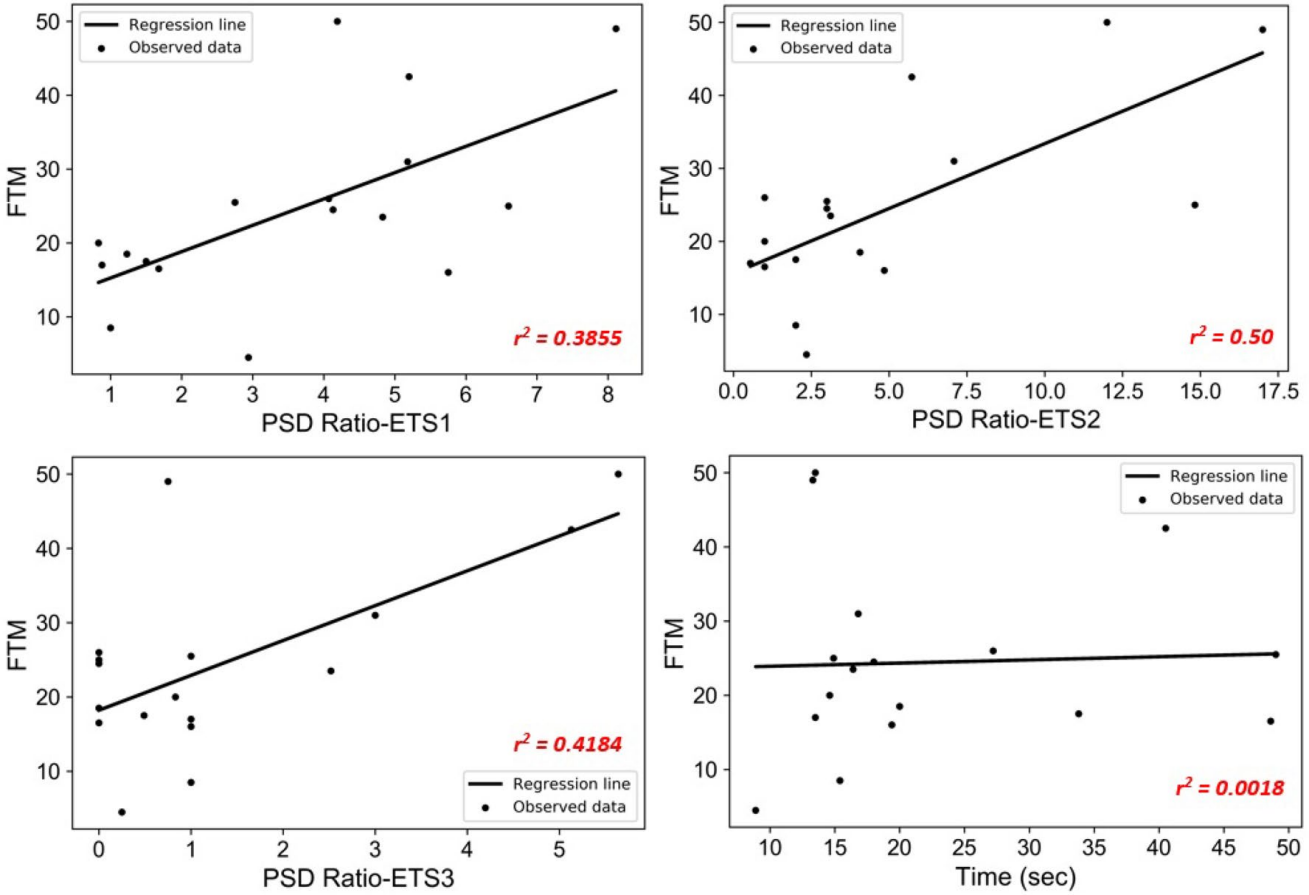
Regression analysis of the PSD ratio versus FTM score at three sensing locations.

Regression analysis was performed to determine the relationship between FTM clinical scores and each sensor's PSD ratio (Fig. 2). The dependent variable is FTM, and the independent variable is the PSD ratio, performed using a total observation of 17 subjects. The coefficient of determination (*r*^2^), Root Mean Square Error (RMSE) and the regression equation for the three sensors are shown in Table 2. The results show that for sensor-1,* r*^2^ = 0.3855; for sensor-2,* r*^2^ = 0.50; and for sensor-3,* r*^2^ = 0.4184*. *This shows that the estimation of FTM was of moderate strength using sensor-2, while it was weak for sensors 1 and 3.

**Table 2:** Regression analysis of PSD ratio vs FTM score at the three sections of the arm.

Regression analysis	Sensor-1 PSD ratio-ETS1 versus FTM score	Sensor-2 PSD ratio- ETS2 versus FTM score	Sensor-3 PSD ratio- ETS3 versus FTM score
Coefficient of determination (r-squared)	0.3855	0.50	0.4184
Root mean square error (RMSE)	9.6476	8.7891	9.3858
Regression equation	11.6747 + (3.5655*PSD ratio)	15.6031 + (1.7763*PSD ratio)	18.2019 + (4.6912*PSD ratio)

Regression analysis between *T* and PSD ratio was very low (*r*^2^ = 0.0905). Being independent of one another yet correlated with tremor severity, these two features are suitable for joint use to estimate the FTM score.

**Figure 3:**
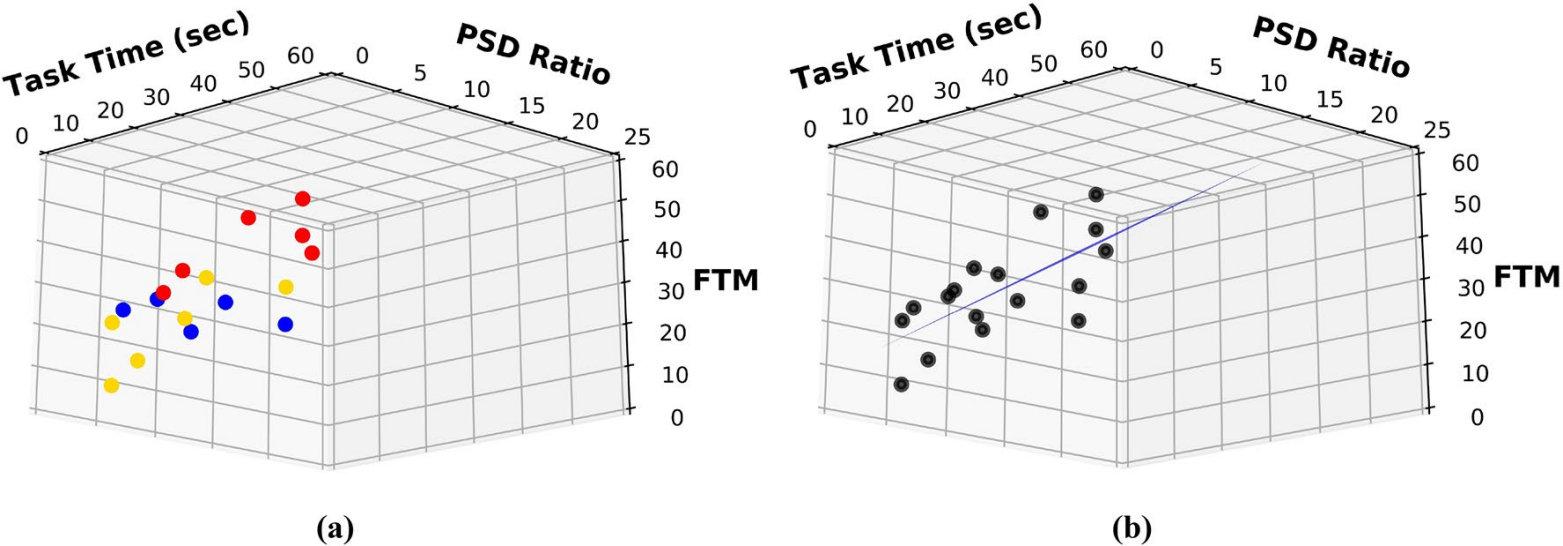
(**a**) Shows the 3D scatter plot of the FTM, PSD Ratio and Task Time representing the groups of ET − 0 (yellow), ET + 1 (blue) and ET + 2 (red), (**b**) Shows the 3D regression plot of the regression Eq. (1).

Regression analysis using the least-squares method was performed to estimate the FTM score from the two sensor features. Figure 3 shows the 3D plot of the parameters used for the regression model. The dependent variable was FTM score, and the two independent variables were *T* and PSD ratio. Data of 17 people with ET were analysed and the regression analysis resulted in a model equation:

1 $$(y \, = \, 8.2439 \, + \, \left( {0.2791*T} \right) \, + \, \left( {1.9890*PSD \, ratio} \right))$$

**Figure 4:**
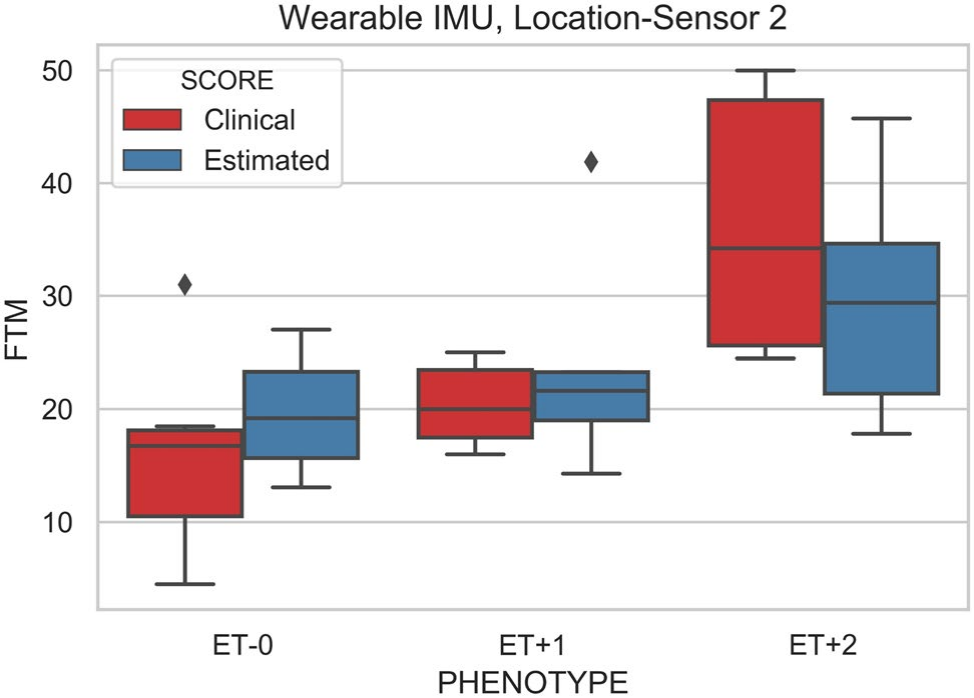
Shows the ET Phenotype of the clinical and estimated FTM scores.

The validation of this model was done by computing the correlation of the model estimates with the clinically-derived FTM scores, as shown in Table 3; *r*^2^ = 0.561 and RMSE = 8.158. Figure 4 shows the comparison between clinically obtained FTM for the three ET subtypes and the FTM estimation from the model (Eq. 1) using wearable sensor data.

**Table 3:** Regression Analysis for the estimation of FTM.

Model	OLS
Coefficient of determination (R-squared)	0.561
Root mean square error (RMSE)	8.158
Regression equation	8.2439 + **(**0.2791*time) + (1.9890*PSD ratio)

### Classification of ET phenotype

A support vector machine (SVM) classifier was applied to the PSD ratio and *T* statistics in two-class and four-class computations. For the binary classification of ET and control, accuracy was 85.71%. The 4-class SVM classifier, comprising ET − 0, ET + 1, ET + 2 and controls, achieved only an accuracy of 57.14% using the combination of *T* and PSD ratio. The predictive power for ET phenotype, according to blinded clinical assessment, was therefore moderate. Table 4 shows the SVM classification accuracy.

**Table 4:** SVM classification accuracy.

		2 Class kernels used	2 Class accuracy in percent (ET and controls)	4 Class kernel used	4 Class accuracy in percent (ET − 0, ET + 1, ET + 2 and controls)
Time	–	RBF	74.28	Linear	54.28
PSD Ratio	–	Linear	85.71	Linear	54.28
Time	PSD Ratio	RBF	82.85	Linear	57.14

## Conclusion

This paper has developed a more accurate method for measurement of ET severity by wearable accelerometer sensors. We determined that, of the three positions tested (Fig. 1b), the most suitable sensor location is on the forearm, at the mid-point between the lateral epicondyle of the humerus and the anatomical snuff box. The PSD 4–12 Hz: 0.5–4 Hz ratio drawn from acceleration data, combined with the total duration of the spiral drawing task, showed a moderate to good correlation (*r*^2^ = 0.561) with clinical FTM scores. While the accuracy of the system to discriminate ET from control was 85%, classification was much poorer when attempting to separate ET subgroups. Between control, ET − 0, ET + 1 and ET + 2 groups, accuracy was only 57%, perhaps reflecting the emphasis on clinical features other than tremor in the Consensus classification scheme. The advantage of this method is that these easy-to-wear, wireless sensors can be used to assist neurologists to measure and to record tremor severity in deciding treatment options for their patients.

## Discussion

This study has confirmed the hypothesis that the ratio of PSD, along with the total time taken to perform the pre-defined Archimedes spiral sketching task, correlates with tremor severity measured clinically by FTM scoring.

### Task execution time

Participants with ET took significantly longer than controls to complete the standardised drawing task. However, the total duration of the activity *T* was found to correlate very weakly (*r*^2^= 0.002) with the FTM score.While there is evidence that ET patients have a degree of slowness of movement^34–38^, this is not captured by the FTM scale, or by other clinical rating scales for ET. Rapid alternating limb movements are performed abnormally slowly^35–37^, and the rhythmicity of repetitive hand movements is impaired by ET^38^. Slowness of movement in ET has, in some studies, been comparable in degree with the bradykinesia of early PD^36^. Both the tremor itself and slowness that is independent of the tremor could therefore contribute to ET patients taking longer to sketch the pre-defined spiral.

### PSD ratio

We used the ratio of PSD to validate the use of sensors for estimating the FTM score data. The PSD ratio determined the relative power in the two bands, obviating the need for normalisation and minimising effects of inter-subject variation. The 0.5—4 Hz band includes mostly voluntary muscle activity in both unimpaired and tremor-impaired individuals, whereas the 4 -12 Hz band includes physiological and pathological tremor. While tremor frequency tends to be a little greater in physiological tremor, there is substantial overlap with ET frequencies^24^. The chief difference is that pathological tremor usually has more power than physiological tremor, so the relative power at the tremor frequency (or even in the entire tremor band) is greater in tremor-impaired individuals. Power in the lower, voluntary band is likely to depend on the activity being performed. There may also be differences between ET and control groups in this band because of slowness of voluntary movement in ET, as discussed in the previous section.

ET has a frequency range of 4–12 Hz^18,31^. Power spectrum analysis of accelerometer signals can discriminate effectively between PD and controls, between ET and controls, and between PD and ET^24^. Previous research has shown the effectiveness of power spectral analysis of acceleration signal is effective in the detection of tremor^24,39–41^. Similar to our analysis, they reported the ratio of the PSD between segments with and without tremor, which was identified manually. None of these studies had investigated the relationship of these measures to the severity of the tremor disorder. Our pilot study showed a weak correlation when using raw PSD, while the PSD ratio, combined with total duration, was established as a reliable feature set to differentiate ET from controls and to estimate FTM score.

### Placement of wearable sensors

Wearable sensors may use accelerometers, gyroscopes, and electromyography for tremor detection. Sensors may be placed at single or multiple body locations. Some previous studies have had limitations in their validation of different sensor locations and estimation of sensor accuracy. A sensor's location can affect the accuracy of detection^22,23,42^. We placed sensors at three sites and identified the most suitable of these locations for tremor measurement. Taken individually, the mid-forearm Sensor-2 performed better than the proximal and distal sensors to estimate tremor severity by PSD ratio. The explanation for this may involve several factors. Spiral drawing involves greater proximal muscle use than writing, though wrist flexion–extension motion produces much of the tracing oscillation that can be seen in Fig. 6^25^. More severe ET tends to be more disseminated^43^, which could contribute to a more proximal tremor emphasis. Device weight, of mobile phones in particular, can also affect long-term monitoring functions^44^, suggesting the need for lightweight sensors instead of smartphones.

### Development of wearable sensors as clinical tools

Previous research using smartphone technology highlights some of the challenges in this area. Episodic rather than continuous data recordings, collected under artificial conditions at certain study intervals, have been reported^45^. The quality and quantity of such information depends on the compliance and motivation of patients^23^. The sensing method proposed in our study is task-specific and employed in a clinical environment that mitigates some of these limitations. To understand the fluctuation of tremor over time, clinicians rely on the accounts of their patients. This can be misleading and suffers from recollection bias^46^. Wearable sensors have advantages of monitoring over long periods and during functional activities, but there is still more work to be done in this area for clinical applications. Another potential advantage of wearable sensing methods is the ability to store a large quantity of data, even though there are limitations in analysing these data sets to provide clinically relevant information^47^. Our study employed a single motor task for the analysis and showed high correlation with a clinical tremor severity scale. Some of our methodology, particularly anatomical sensor placement, could be extended to long-term ambulatory monitoring of the effects of ET on daily living. In the future, both types of applications are likely to find a place in clinical management and clinical trials.

## ET and ET plus

The Consensus scheme attempts to identify clinical markers that may correspond to underlying pathophysiology. The sub-classification of the disorder into ET and ET plus is based on the presence of additional clinical features, the majority of which are unrelated to tremor. While criteria for ET plus do not include tremor severity, we found significantly higher FTM scores in ET plus participants, highlighting a possible weakness. Critics of the consensus statement point out that the ET plus concept may be insensitive to biological differences because its criteria tend to select for more prolonged and severe ET^7^. Our automated classification into ET − 0, ET+1, and ET+2 showed only a modest accuracy of 57.14%. The measurements performed better in predicting the clinical FTM score, which is the novel finding of this study of wearable technology in ET.

## Limitations of this study

This study has two major limitations, which concern repeatability and sample size. Each participant was investigated only once and hence the repeatability was not tested. The sample size was not large enough to perform a detailed analysis of the differences within ET subgroups.

## Methodology

### Participants and clinical assessments

Ten men and nine women with a clinical diagnosis of ET were recruited from the Movement Disorders service at Monash Health. Their mean age was 67.2 ± 13.0 and the mean duration of tremor symptoms was 21.7 ± 19.0 years. All complied with the Axis 1 definition of ET in the 2018 Consensus Statement on the Classification of Tremors^6^. No participant with ET met any of the Axis 1 exclusion criteria for ET and ET plus. Twenty age-matched healthy participants acted as controls for spiral drawing tasks. Two ET and 2 control participants were excluded from the analysis because of noisy or incomplete IMU data recording.

A structured interview of ET subjects concentrated on clinical aspects of the tremor disorder. A MoCA was also conducted^48^. Two movement disorder neurologists who were blinded to clinical information scored the FTM^28^ from videotapes. Mean total scores were obtained for each subject. The blinded assessors then classified the ET disorder as defined in Axis 1 of the Consensus Statement. They were also provided with a summary of the structured clinical interview, the MoCA results, and videotapes of testing of goal-directed limb coordination, distal limb rapid alternating movement and gait (normal and tandem). Subjects were classified as ET plus by the presence of any of the following features: impaired tandem gait, questionable dystonic posturing, memory impairment, mildly impaired goal-directed incoordination of unknown significance, mildly impaired rapid alternating movement of unknown significance, tremor at rest. For each participant, a neurologist familiar with the case also phenotyped the tremor disorder from the videotape. A majority classification was then obtained on the presence or absence of plus features and on the number of plus features documented. The study was conducted following the human experiments Helsinki Declaration (revised 2004) and approved by the Monash Health and RMIT University Human Research Ethics Committees (HREC Project Number: 184981). All participants in this study gave their written informed consent before data recording.

### Equipment and data recording

Wearable IMU sensors (Delsys Trigno, USA) with an inbuilt three-axis accelerometer, gyroscope, magnetometer, and electromyography were used for data recording. Only the accelerometer data is reported in this paper, which concentrates on the use of this modality to analyse tremor as outlined in previous research^39,49^.

Sensors were placed at three points on the dominant arm:

- Sensor-1 at the mid-point between the styloid process of the ulna and the head of the third metacarpal;
- Sensor-2 at the mid-point between the lateral epicondyle of the humerus and the anatomical snuff box;
- Sensor-3 at the mid-point between the lateral tip of the acromion process and the lateral epicondyle of the humerus.

**Figure 5:**
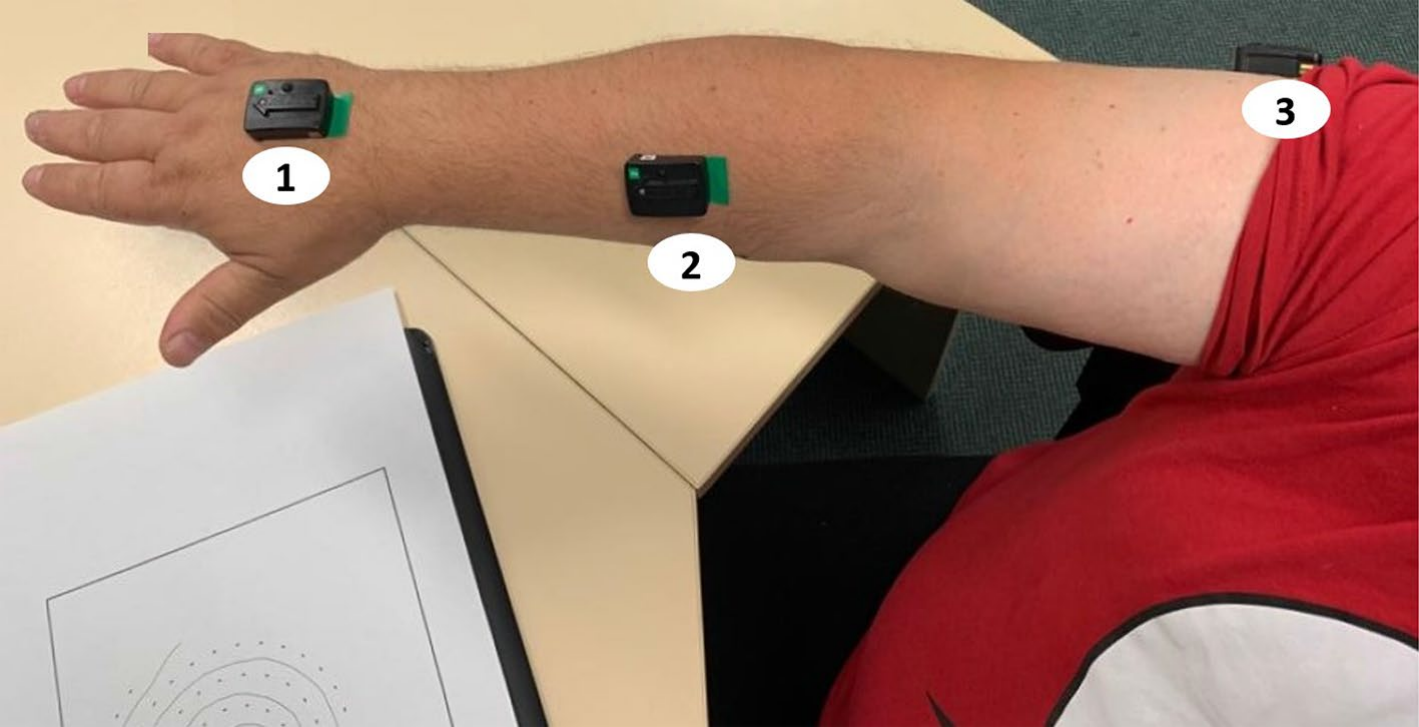
Participant performing the spiral drawing task with the wearable sensors mounted on the upper limb.

**Figure 6:**
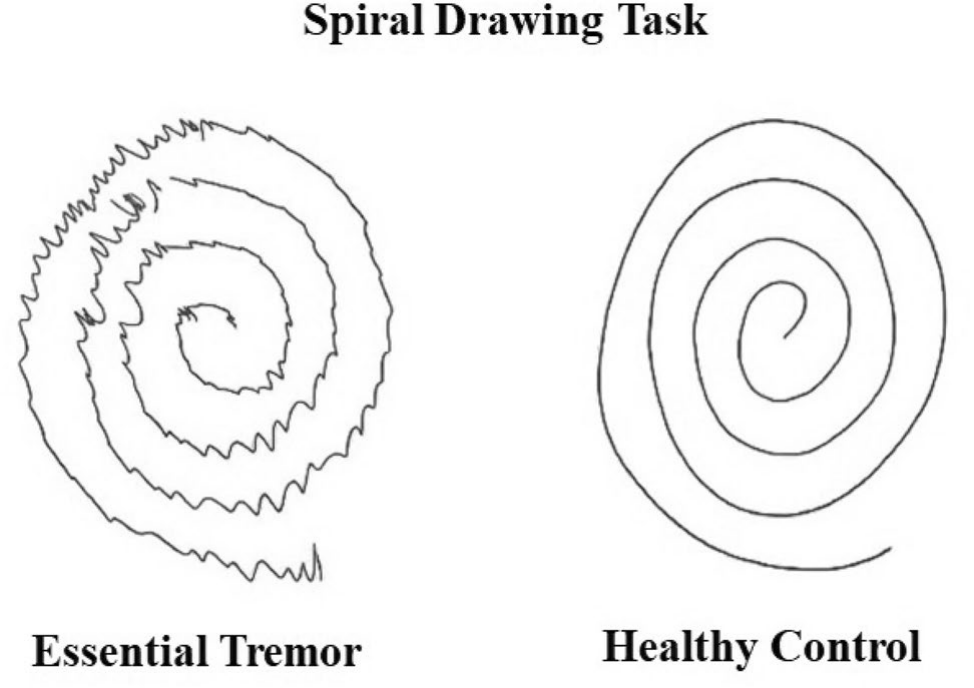
Sketch of the spiral drawing task performed by both control and ET patient.

Figure 5 shows the positioning of wearable sensors. Participants were recorded while drawing on a digital tablet (Wacom Intuos Pro Large, A3 sized) with a pressure-sensor mounted ink-pen. The tablet was overlaid with a sheet of paper to normalise the experience as much as possible. It was set upon a standard height desk, positioned as was most comfortable to each participant. They were asked to draw the spiral at their convenient speed. Customized software was developed in *c-sharp*, which integrated the digital tablet and Delsys Trigno IMU signals. Pen-tip pressure was used to separate movement on and above the tablet. Pen movements with pressure = 0 were labelled as ‘pen-up’ strokes, a ‘pen-down’ stoke was any movement while pressure was > 0. The acceleration data was recorded at 148.1 samples/sec and stored in a .csv file format. Figure 6 shows the ink-pen record of the spiral drawing task performed by ET and control participants.

Previous researchers into functional neuroimaging^50,51^, timed clinical motor assessments^52,53^, and activity monitoring during daily living conditions^23,54–57^ have faced choices about motor task, the duration of recording, and other extrinsic factors that may influence the outcomes. We adopted a standardised task of spiral sketching, which is routinely used in clinical practice, to minimise some of these potential limitations.

### Data analysis

Acceleration data were recorded from the three IMUs while participants drew the pre-defined spiral. Data was segmented from the start to the end of the task from the tablet's pen-up and pen-down. To overcome the problem of the variation of the angle of placement of the sensor, vector magnitude was derived from the raw triaxial accelerometer data as $\sqrt{{x}^{2}+{y}^{2}+{z}^{2}}$^22,58^. This was filtered using a high pass filter of 0.5 Hz to remove artefacts.

People with ET have heightened activity in the frequency range of 4–12 Hz. In healthy people, physiological tremor produces much less power in this range and the 0–4 Hz band is dominant^20,59^. While there can be inter-subject variability, this study proposes the ratio of PSD between these two frequency ranges as a feature likely to be higher in ET than controls. From the three-axis acceleration data, the vector magnitude was calculated, then the PSD ratio was calculated from the ratio of the PSD at 4–12 Hz to the PSD at 0.5—4 Hz.

People with ET take longer to perform comparable writing tasks ^60^. This could result from the tremor itself, or from the slowness of voluntary movement that also occurs in ET^7,36^. The time feature *T,* corresponding to the pen-down duration for the whole task, was measured and evaluated against the FTM.

### Statistical analysis

Shapiro–Wilk testing, conducted to check for normality distribution of drawing parameters^61^, showed a non-Gaussian distribution. Statistical analysis was performed using the non-parametric Mann–Whitney U tests to determine the significance of group differences. Analyses were conducted on differences in ET and controls parameter values. Kruskal–Wallis test was performed to determine p-values between the different groups.

### Regression analysis

Linear regression analysis using the least square method were performed to determine the strength of relationship between the sensor parameters and the clinically derived FTM score. From the 17 ET and 18 healthy controls who participated in the study, the PSD ratio and the task time were the sensor parameters used as independent variables, and the mean FTM scores by clinical assessment were the dependent variables. Inter-rater assessment was done by correlation analysis on the FTM score generated by the two independent and blinded neurologists. The coefficient of determination r^2^ values are obtained from the analysis.

### Classification

An SVM classifier was applied to classify individually and in combination the PSD ratio and total task time for multiclass problems comprising control and ET subgroups. The analysis was based on the Leave-One-Out Cross-Validation (LOOCV) technique using linear and radial basis function kernels, and the best classification accuracy was chosen from these two kernels. In this cross-validation technique, the number of folds equals the number of instances in the data set. Thus, the algorithm applies once for each instance, using all other instances as a training set and the selected instance as a single-item test set. The result is computed by taking the mean of individual evaluations. Studies have shown LOOCV to be a more reliable performance for similar size datasets^62^. The inputs are the PSD ratio measured from the IMU sensor unit and the duration of each instance from the 17 ET and 18 healthy control participants. All computation, including statistical analysis, was performed using Python 3.8.
